# Trends and characteristics of hospitalisations from the harmful use of opioids in England between 2008 and 2018: Population-based retrospective cohort study

**DOI:** 10.1177/01410768221077360

**Published:** 2022-02-03

**Authors:** Rocco Friebel, Laia Maynou

**Affiliations:** 1 Department of Health Policy, The London School of Economics and Political Science, London, Houghton Street, WC2A 2AE, UK; 2 Center for Global Development Europe, London, Abbey Gardens, SW1P 3SE, UK; 3 Department of Econometrics, Statistics and Applied Economics, Universitat de Barcelona, 08034 Barcelona, Spain; 4 Center for Research in Health and Economics, University of Pompeu Fabra, 08005 Barcelona, Spain

**Keywords:** Population trends, drug misuse, health policy, poisoning

## Abstract

**Objective:**

To examine the trends and characteristics of opioid-related hospital admissions in England over 10 years, and its burden for the National Health Service and public finances.

**Design:**

Patient-level data from the Hospital Episode Statistics database to examine all opioid-related hospitalisations from 2008 to 2018, stratified by type of opioid admission and patient demographics.

**Setting:**

All National Health Service hospitals in England.

**Participants:**

Patients hospitalised from the harmful use of opioids.

**Main outcome measures:**

The number of opioid-related hospitalisations, length of stay, in-hospital mortality, 30-day readmission rate and treatment costs.

**Results:**

Opioid-related hospitalisations increased by 48.9%, from 10,805 admissions in 2008 to 16,091 admissions in 2018, with total treatment costs of £137 million. The growth in opioid-related hospitalisations was 21% above the corresponding rate for all other emergency admissions in England. Relative changes showed that hospitalisations increased most for individuals older than 55 years (160%), those living in the most affluent areas of England (93.8%), and suffering from four co-morbidities (627.6%) or more. Hospitals reduced mean patient length of stay from 2.8 days to 1.1 days over 10 years. Mean in-hospital mortality was 0.4% and mean 30-day readmission risk was 16.6%.

**Conclusion:**

Opioid use is an increasing public health concern in England, though hospitalisation and mortality rates are less pronounced than in other countries. There are concerns about significant rises in hospitalisations from older, less deprived and sicker population groups. Our findings should prompt policymakers to go beyond monitoring mortality statistics when assessing the impacts of harmful use of opioids.

## Introduction

Every year more than 500,000 people die from a drug overdose worldwide,^1^ out of which 70% are linked to the harmful use of opioids,^2^ including prescription opioids such as tramadol, and illicit opioids such as heroin. While the United States (USA) remains at the centre of the opioid crisis with approximately 130 deaths daily,^3–6^ between 2011 and 2016 opioid-related mortality has seen increases by an average 20% across developed nations.^7^ In England, more than half of all drug-related deaths are caused by opioids, although annual mortality has stabilised at around 2000 opioid-related deaths in recent years.^8^ Possible reasons for this trend may include current strategies to widened availability of naloxone,^9^ an overdose-reversal drug that can help stabilise respiratory function.^10^ However, while much focus has been given to official opioid mortality statistics,^11^ they likely discount the true burden of harmful opioid use, particularly if linked to frequent interactions with the health care system.

Latest government estimates predicted 261,294 high-risk opioid users, or 7.4 users per 1000 population aged 15 to 64 years in England.^8,12^ Despite the salience of this issue, little evidence exists on users’ demographics, socioeconomic characteristics and regional distribution, their interactions with the healthcare system and characteristics of their hospital stays. A large proportion of individuals who overdose on opioids frequently require treatment as a hospital inpatient. While more than 7000 people are treated in hospitals for opioid use in the USA every day,^13^ the number of opioid-related hospitalisations for the English National Health Service and the costs for the taxpayer are unknown.

We analysed hospital admissions related to opioid overdoses for all National Health Service hospitals from 2008 to 2018, using health records for all patients in the National Health Service. By examining health services use in inpatient settings, emergency readmission rates, patient characteristics and the cost of treatment, this study provides evidence on the scale of harmful use of opioids in England beyond official mortality statistics, which can help direct the focus of strategies aiming to address the opioid epidemic.

## Methods

### Study cohort

Access to the Hospital Episode Statistics (HES) database was obtained from National Health Service Digital (i.e*.* the non-departmental public body responsible for information, data and Information Technology systems in England) for the period from 2008 to 2018. This national administrative database contains pseudonymised and unidentifiable information on all hospital inpatients accessing care in the English National Health Service from 1989. Patient information includes demographics (e.g*.* age, gender, ethnicity and socio economic status), diagnosis, treatment and in-hospital death. HES data are recorded in finished episodes of care, which relates to the clinician responsible for the respective aspect of care. To avoid multiple counting, we linked episodes from patient admission to discharge into spells based on the unique patient identifier, which also accounted for transfers to another provider if part of the same treatment plan. To standardise the number of hospital admissions per 1,000,000 population, we obtained annual population statistics from the Office for National Statistics (i.e*.* a non-government department providing statistical services to the United Kingdom parliament).

We defined unplanned (non-elective) hospitalisations due to opioids based on the primary diagnosis codes recorded according to the International Statistical Classification of Diseases and Related Health Problems, 10th edition. The selection of codes followed those used by the Office for National Statistics to collate official opioid-related mortality statistics (see supplementary Appendix A). This included hospitalisations for opioid dependence, non-dependent harmful opioid use and opioid poisoning due to opium, heroin, methadone and other related opioids and narcotics. The same codes have been used in previous studies that investigated opioid emergency hospitalisations based on insurance claims data in the USA.^13,14^

To compare changes in population-standardised, opioid-related hospitalisation to changes in non-opioid–related hospital admissions, we obtained information from hospitalisations due to all other causes (i.e*.* exclusive of opioid-related hospitalisations), alcohol consumption and all other illegal drugs, as classified by major diagnostic categories according to International Statistical Classification of Diseases and Related Health Problems chapters.

### Study outcomes

The primary outcomes in this study were the number of opioid-related hospitalisations and their associated in-hospital mortality. For each financial year, we calculated the crude number of hospitalisations and the population-standardised number of hospitalisations expressed per 1,000,000 population. We identified patients who died during their hospital stay based on the discharge method recorded in HES and calculated mortality rates as the ratio of total number of expired patients divided by the total number of opioid-related hospitalisations in each financial year.

The secondary outcomes studied are the patients’ length of stay and number of total bed days, the observed readmission rate within 30 days and the treatment costs. Length of stay was calculated as the difference between day of admission and day of discharge. Patients who were admitted and discharged on the same day, or without staying overnight were recorded with a zero length of stay. While it is possible that patients admitted due to an opioid overdose remain in hospital for several days, mortality risk is likely highest on the same day as the admission. We therefore calculated the total number of bed days per year as the sum of the overall length of stay plus the total number of patients recorded with zero length of stay, which accounted for the relative health service use and associated costs from such patients. Readmission rates refer to all-cause, unplanned admissions occurring within 30 days from discharge following an index admission. This definition is consistent with national guidelines used under the English national readmission reduction programme since 2011.^15^ The treatment cost per patient spell was calculated by applying the Healthcare Resources Groups software to diagnosis, procedure and patient characteristics recorded in HES.^16^ The programme classified patients to case-mix groups and levied payments according to the national tariff 2017/18. Combining the payments of all patients for every year allowed us to investigate the total cost for treatment of opioid-related hospitalisations over time. To our knowledge, this is the first study to estimate healthcare costs due to the harmful use of opioids in England.

We stratify patients into several demographic groups. For age, we use 0–14 years, 15–24 years, 25–34 years, 35–44 years, 45–54 years, 55–64 years, 65-74 years, 75-84 years, and 85 years or older. For gender, female and male was based on phenotypical classifications. We used the Charlson Comorbidity Index as a measure for patient complexity based on the number of comorbidities recorded in HES. This index is widely used for risk stratification in health services research and was calculated based on the primary and secondary diagnosis codes recorded at the index admission.^17^ We included patient groups with 0 co-morbidities to six or more co-morbidities, respectively. To measure the patients’ level of socio-economic deprivation, we refer to the Index of Multiple Deprivation, which is based on small geographic areas of the patients’ place of residence with on average 1500 residents. The Index of Multiple Deprivation score uses information from the 2015 census and is a composite of seven dimensions that reflect unmet population need. We divided Index of Multiple Deprivation scores into quintiles (i.e. assigning quintile 1 for patients living in most deprived areas to quintile 5 for patients living in least deprived areas) and patients were assigned according to their location in the Index of Multiple Deprivation score distribution.

### Statistical analysis

Descriptive statistics were used to summarise opioid-related hospitalisations expressed per 1,000,000 population and their associated in-hospital mortality rates. We used a multivariable patient-level logistic regression model to examine the relationship between patient characteristics and in-hospital mortality and 30-day readmission risk. To examine the relationship with length of stay, we employed a multivariable patient-level linear regression model with ordinary least squares.^18^ We assigned year fixed effects across all models and hospital fixed effects (except for in-hospital mortality, where the low number of deaths, or lack thereof, resulted in the exclusion of hospitals). We report *p*-values with 0.05 considered as threshold for statistical significance. All analyses were performed using STATA SE 16.

## Results

### Opioid-related hospitalisations

**Figure 1.**
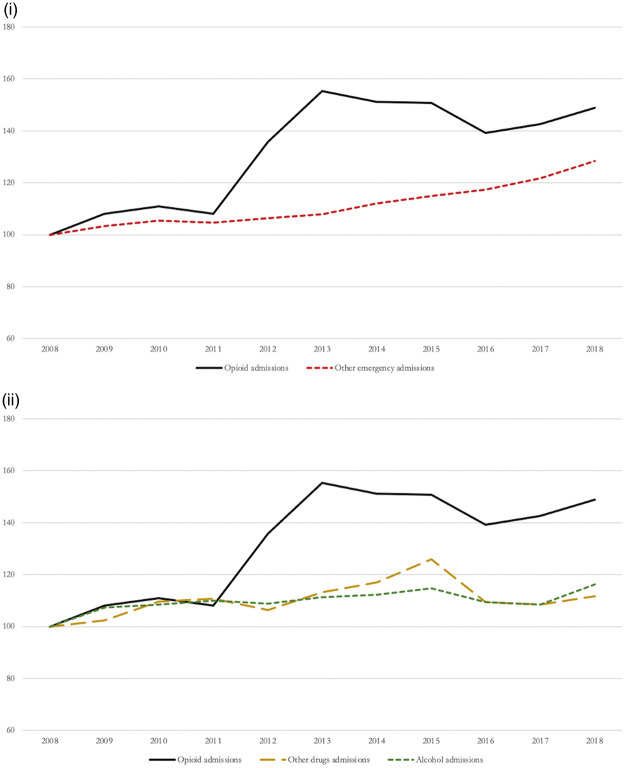
Relative changes in opioid-related admissions compared with other emergency admissions (index 2008 = 100) (i – other emergency admissions; ii – other drugs and alcohol admissions).

Over a 10-year study period, we identified a total of 156,773 opioid-related hospital admissions in England. These increased by 48.9%, from 10,805 admissions (20.85 per 1,000,000 population) in 2008, to 16,091 admissions (28.75 per 1,000,000 population) in 2018 (see Figure 1). In comparison, emergency admissions due to all other causes increased by 28.4%, from 4,999,865 admissions (9649.3 per 1,000,000 population), to 6,421,868 (11,472.3 per 1,000,000 population) between 2008 to 2018, respectively. Moreover, across the 10-year study period, emergency hospital admissions due to other illegal drugs increased by 11.6% and due to alcohol consumption increased by 16.2%.

**Figure 2.**
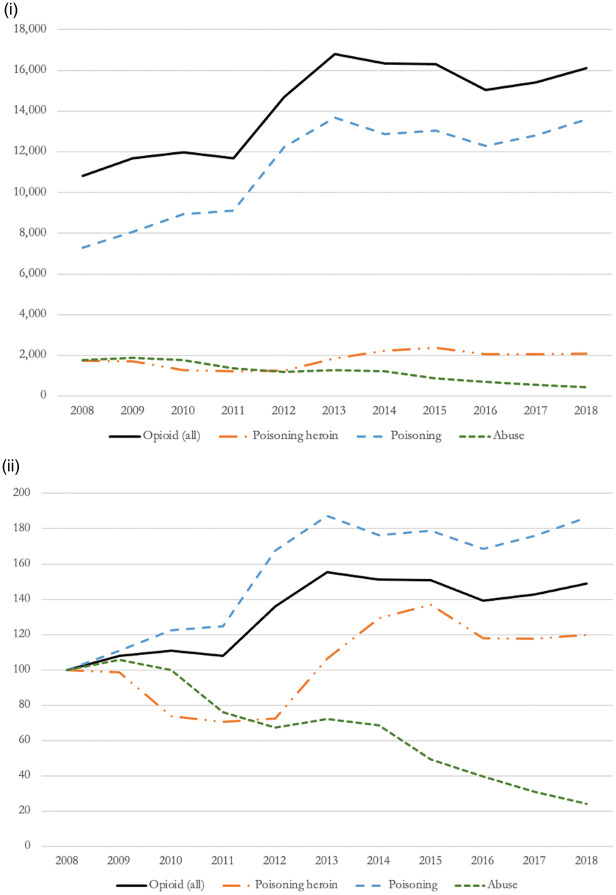
Opioid-related admissions disaggregated by type (i – absolute changes; ii – relative changes index 2008 = 100).

When disaggregating by type of opioid-related hospitalisation based on International Statistical Classification of Diseases and Related Health Problems codes (Figure 2) and taking International Statistical Classification of Diseases and Related Health Problems groupings as an indicator of use severity, we observed a shift towards more severe forms of harmful use of opioids. While the proportion of patients experiencing admissions due to opioid abuse decreased by 76%, from 1778 admissions in 2008, to 430 admissions in 2018, admissions due to opioid poisoning (i.e*.* excluding heroin poisoning) increased by 86%, from 7291 admissions in 2008, to 13,580 admissions in 2018. Approximately one in 10 opioid-related hospitalisations was caused by opioid poisoning from heroin, with relative changes showing an increase of 19.9% compared with 2008.

### Decomposition of opioid-related hospital admissions

**Figure 3.**
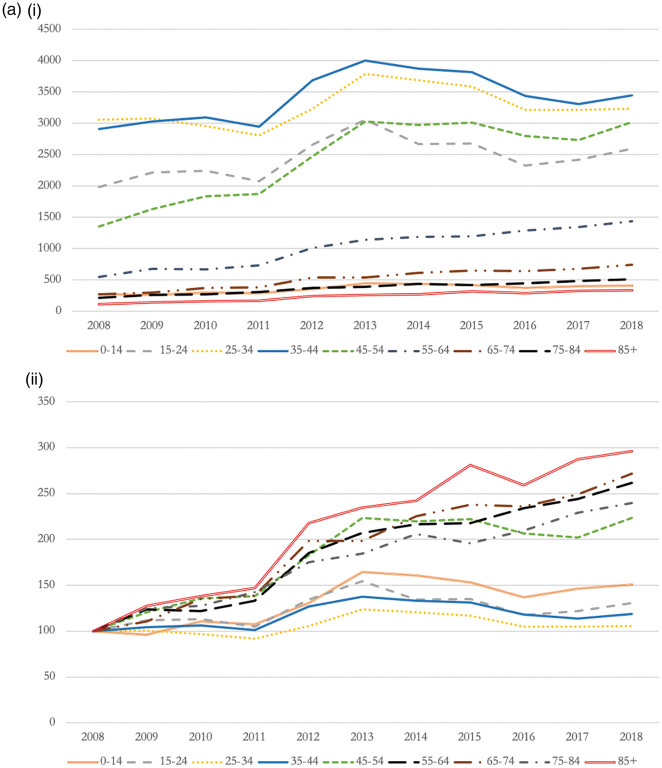

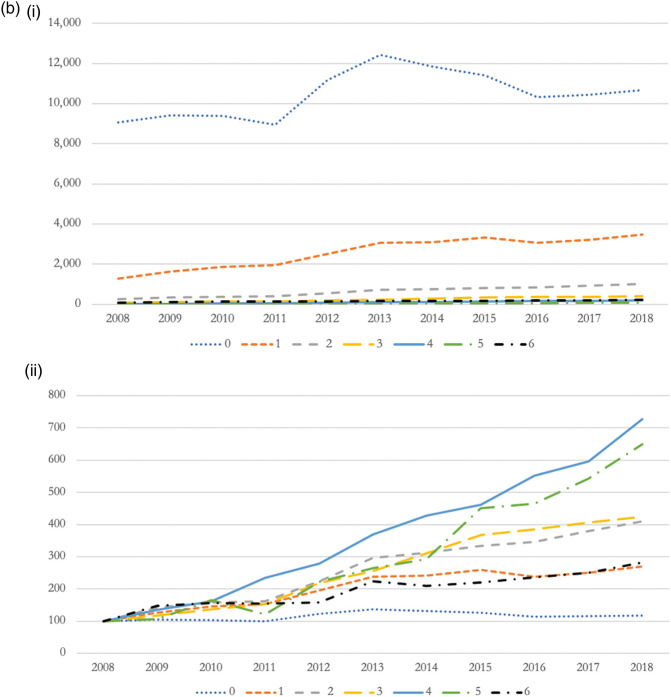

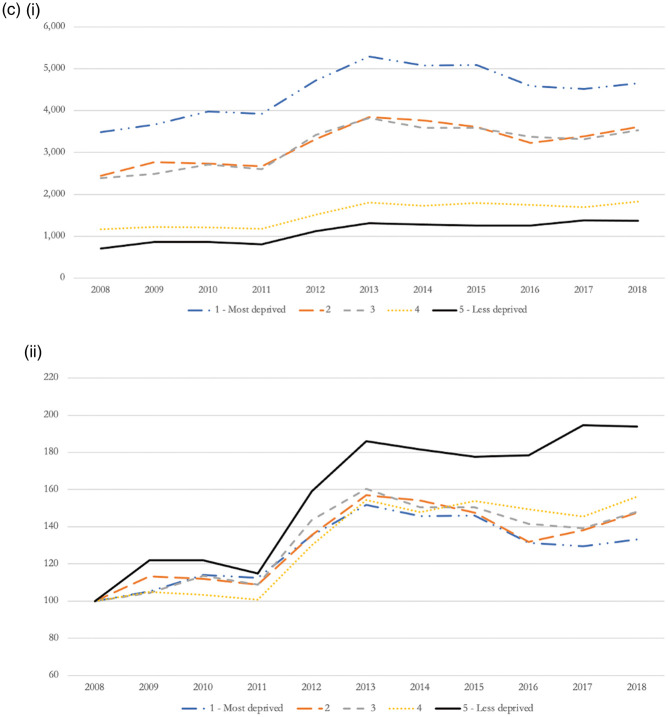
(a) Opioid-related admissions by age group (i – absolute changes; ii – relative changes index 2008 = 100). (b) Opioid-related admissions by Charlson Comorbidity Index (i – absolute changes; ii – relative changes index 2008 = 100). (c) Opioid-related admissions by IMD score (i – absolute changes; ii – relative changes index 2008 = 100).

Opioid-related hospitalisations were evenly distributed among both sexes (49.7% male), and hospitalised patients had a mean age of 39.1 years (see Table 1). While the number of hospitalisations for patients aged below 44 years remained relatively stable across the study period, utilisation patterns from patients aged over 45 years increased significantly. For example, NHS hospitals saw a rise in opioid-related admissions from patients above 55 years by more than 160% over a 10-year period (i.e*.*, 1.5-fold increase in admissions) (see Figure 3(a)).

**Table 1.** Descriptive summary statistics across opioid-linked hospitalisations.

	N (observations)	Mean/proportion
Sex (=1 Male)	156,773	49.72%
Age (years)	154,862	39.07
Age 0–14	154,862	2.58%
Age 15–24	154,862	17.39%
Age 25–34	154,862	23.16%
Age 35–44	154,862	24.26%
Age 45–54	154,862	17.27%
Age 55–64	154,862	7.27%
Age 65–74	154,862	3.70%
Age 75–84	154,862	2.70%
Age 85+	154,862	1.70%
Charlson Comorbidity Index = 0	156,773	73.42%
Charlson Comorbidity Index = 1	156,773	18.19%
Charlson Comorbidity Index = 2	156,773	4.48%
Charlson Comorbidity Index = 3	156,773	1.74%
Charlson Comorbidity Index = 4	156,773	0.75%
Charlson Comorbidity Index = 5	156,773	0.30%
Charlson Comorbidity Index = 6+	156,773	1.13%
IMD quintile 1 – Most deprived	148,225	33.05%
IMD quintile 2	148,225	23.86%
IMD quintile 3	148,225	23.49%
IMD quintile 4	148,225	11.38%
IMD quintile 5 – Less deprived	148,225	8.23%
Number previous admissions (count)	156,773	0.44
Costs (£)	155,596	878.53
*Outcomes*		
* In-hospital mortality (all)*	156,773	0.38%
In-hospital mortality poisoning heroin	19,857	0.91%
In-hospital mortality poisoning	123,860	0.34%
In-hospital mortality abuse	13,056	0.03%
*Length of stay (all)*	155,596	1 (1)^a^
Length of stay poisoning heroin	19,719	0 (1)^a^
Length of stay poisoning	122,874	0 (1)^a^
Length of stay abuse	13,003	10 (12)^a^
* 30-day readmission (all)*	156,773	16.78%
30-day readmission poisoning heroin	19,857	16.29%
30-day readmission poisoning	123,860	17.51%
30-day readmission abuse	13,056	10.55%

Note*:* N denotes the number of observations for each variable, which may vary because of missing values. The maximum N in our sample is 156,773.

^a^Median and interquartile range (in parenthesis) reported.

Most opioid-related hospitalisations (i.e*.* 73.4%) occurred in patients with no underlying co-morbidities (see Table 1). However, we found significant rises in the proportion of hospital admissions from people suffering from multi-morbidity. For example, opioid-related admissions from patients reporting four co-morbidities increased by 627.6% (i.e*.* >6-fold increase in admissions), and increased by 550.0% (i.e*.* 5.5-fold increase in admissions) for patients reporting five co-morbidities (see Figure 3(b)).

We observed a socioeconomic gradient in the relationship between opioid use and hospitalisation rates. Patients living in the most deprived areas were at a four fold increased risk to be admitted because of harmful use of opioids than patients living in the least deprived areas of England (see Table 1). While admissions increased across all Index of Multiple Deprivation quintiles, National Health Service hospitals experienced largest relative rises in opioid-related hospitalisations from patients living in the least deprived neighbourhoods (i.e*.* 93.8%, or one-fold increase in admissions) (see Figure 3(c)).

### Patient outcomes

Mean mortality risk for patients admitted due to opioids was 0.4%; lowest for patients admitted for opioid abuse (i.e*.* 0.03%) and highest for patients admitted for heroin poisoning (i.e*.* 0.9%) (see Table 1). A total of 601 patients died in hospital after admission due to the harmful use of opioids across 10 years. Mean length of stay was 1.8 days but NHS hospitals reduced mean length of stay from 2.76 days in 2008 to 1.12 days in 2018. Because Length of Stay is skewed towards 0, Table 1 reports the median of this outcome, which is equal to 1 day. The mean 30-day readmission rate for patients discharged following an opioid-related hospitalisation was 16.7%; increasing from 14.5% in 2008, to 17.9% in 2018. On average, 30-day readmission risk was 6.3 percentage points lower for patients admitted for opioid abuse compared with poisoning, inclusive and exclusive of heroin poisoning.

Regression output showing the association between patient outcomes and patient characteristics is presented in Table 2. Results show that patient characteristics (i.e*.* sex, age, Charlson Comorbidity Index, Index of Multiple Deprivation and the number of previous admissions) are associated with in-hospital mortality, Length of Stay and 30-day readmissions. Being older and sicker is related to a higher probability of dying, longer length of stay and a higher probability of being readmitted within 30-days after discharge. Findings from repeated regression analysis for the three outcomes per year, show consistent associations for age and Charlson Comorbidity Index (supplementary Appendix B).

**Table 2.** Regression output of the association between patient characteristics and outcomes.

	(1)	(2)	(3)
	Logistic regression	OLS	Logistic regression
	In-hospital mortality	LoS	30-day readmission
Female	Reference	Reference	Reference
Male	1.736***	−0.052***	1.052***
	(0.153)	(0.015)	(0.015)
Age 0–14	Reference	Reference	Reference
Age 15–24	2.108	−0.235***	1.861***
	(2.178)	(0.026)	(0.120)
Age 25–34	7.264**	−0.185***	2.066***
	(7.314)	(0.028)	(0.133)
Age 35–44	8.727**	−0.071**	2.189***
	(8.785)	(0.029)	(0.141)
Age 45–54	8.200**	0.003	2.160***
	(8.284)	(0.030)	(0.140)
Age 55–64	12.74**	0.243***	2.148***
	(12.91)	(0.037)	(0.146)
Age 65–74	15.41***	0.595***	2.172***
	(15.68)	(0.056)	(0.157)
Age 75–84	28.93***	1.124***	2.312***
	(29.41)	(0.083)	(0.175)
Age 85+	28.77***	1.341***	2.412***
	(29.35)	(0.119)	(0.198)
Charlson Comorbidity Index = 0	Reference	Reference	Reference
Charlson Comorbidity Index = 1	2.363***	0.206***	1.352***
	(0.286)	(0.020)	(0.025)
Charlson Comorbidity Index = 2	3.472***	0.399***	1.781***
	(0.551)	(0.049)	(0.059)
Charlson Comorbidity Index = 3	5.499***	0.683***	2.046***
	(1.044)	(0.092)	(0.099)
Charlson Comorbidity Index = 4	5.303***	1.064***	2.232***
	(1.277)	(0.155)	(0.157)
Charlson Comorbidity Index = 5	7.452***	1.245***	2.380***
	(2.253)	(0.267)	(0.260)
Charlson Comorbidity Index = 6+	9.217***	0.648***	1.893***
	(1.687)	(0.118)	(0.124)
IMD quintile 5 – Most deprived	0.852	−0.069**	1.161***
	(0.133)	(0.030)	(0.035)
IMD quintile 4	0.963	−0.072**	1.126***
	(0.152)	(0.031)	(0.035)
IMD quintile 3	0.942	−0.077**	1.121***
	(0.151)	(0.031)	(0.035)
IMD quintile 2	1.171	−0.021	1.020
	(0.194)	(0.034)	(0.035)
IMD quintile 1 – Less deprived	Reference	Reference	Reference
Number of previous admissions	0.740***	−0.018***	1.372***
	(0.048)	(0.003)	(0.012)
N	148,213	146,581	148,054
Pseudo R-squared or R-squared	0.116	0.524	0.056
Hospital FE	No	Yes	Yes
Year FE	Yes	Yes	Yes
Years	2008–2018	2008–2018	2008–2018

Note: Significance levels: ***p<0.01, **p<0.05, *p<0.1; Models 1 and 3 (logistic regression): odds ratios reported; Model 2 (OLS: ordinary least squares): marginal effects reported; and standard errors reported in parenthesis. In Model 2, patients who died were excluded for our analysis (601).

### Cost of opioid-related hospitalisations to the English National Health Service

The total treatment cost of opioid-related hospitalisations for the study period was £137 million. The cost in 2008 was £12.4 million and £10.7 million in 2018, showing a decrease of £1.7 million (13.7%). The reduction in cost is likely related to a decrease in Length of Stay. The average cost per patient admissions also reduced from £1,158 to £671, from 2008 to 2018, respectively. Moreover, we assessed the relative burden of opioid-related hospitalisations on National Health Service hospitals through the number of bed days, which correlates with total cost. Across the 10-year study period, patients admitted due to the harmful use of opioids accounted for 359,983 total bed days, with the annual number of bed days reducing from 34,255 bed days in 2008, to 26,175 bed days in 2018. This reduction was achieved by hospitals even though the number of opioid-related hospitalisations had increased.

## Discussion

There are an estimated 261,294 high-risk opioid users aged 15 to 64 years in England.^8^ Death caused by an overdose in this group has risen by more than 20% between 2011 and 2016,^7^ but incidences have since stabilised at around 2000 deaths per year.^19^ This could be attributed to combined efforts of non-government organisations, local providers and payers of care, and national policymakers in pursuing strategies aimed at reversing trends of opioid-related mortality,^20^ for example through increasing community access to the overdose reversal drug naloxone. However, mortality statistics likely mask an important aspect of harmful opioid use nationally, as most high-risk individuals frequently require access to hospital services. Indeed, we found that opioid-related hospitalisations have increased by more than 48.9% over a 10-year period, from 10,805 admissions in 2008 to 16,091 admissions in 2018, with a total treatment cost of £137 million incurred by the NHS.

To our knowledge this is the first study to explore the evolution of opioid-related mortality in England through examining the volume of health services use and patient characteristics. Similar to work in the USA,^14^ we found hospital admissions in England concentrated among individuals aged 25 to 44 years and admission risk increased in line with levels of deprivation.^21,22,28^ However, in relative terms, hospital admissions for people aged 55 years and older have increased by 160% across the study period, and with those from the least deprived areas showing a 93.8% increase in admissions compared with 2008. Our study was not able to unfound fears about adverse effects from long-term use of prescription opioids in patients combating cancer pain or chronic pain.^23^ Even though the majority of patients treated for opioid abuse in English hospitals had no underlying co-morbidity, we observed a 627.6% increase in admissions for people with four comorbidities, or more.

Despite increases in the severity type of opioid use, reductions in Length of Stay possibly relates to advances in hospital treatments, notably the widespread use of opioid antagonists. However, patient risk for an all-cause, emergency readmission within 30 days increased from 14.4% to 17.8%, which is significantly above the national average readmission rate of 6.6%.^15^ Findings from our analysis of patient level, hospital data highlighted the need to improve targeting of programmes designed to prevent future harmful use of opioids among high-risk individuals.

### Strengths and limitations

Our analysis was based on administrative hospital data and is subject to residual error resulting from misclassification and changes in coding practices over time. However, HES data are generally considered of high quality, as they are derived from data used for hospital reimbursement and have been used in the study of adverse drug reactions,^24^ and policy evaluations linked to other patient groups.^25,26^ The focus on hospital inpatients may have missed some patients treated in the Accident and Emergency Departments who did not get admitted as an inpatient, therefore providing conservative estimates of the true burden to the National Health Service.

To identify opioid-related hospitalisations, we used the primary diagnosis rather than all diagnosis fields. It is possible that in geographical areas that experienced higher rates of harmful use of opioids, greater awareness among clinicians may be reflected in their coding practices.^13^ However, our identification method was considered to be a more meaningful approach to examine causes of admission and was found to be more sensitive to detect mortality risk among opioid patients.^27^

Because our data were not linked to death registries, we were not able to calculate patient risk of death outside the hospital environment. However, we were able to show that on average, fewer than 60 individuals admitted because of opioids died in hospital every year, accounting for 3% of the annual total death count recorded in the official Office for National Statistics statistics. This finding supports the notion for improving access to emergency medical treatments in the community to protect against the mortality risk from opioids and accentuates the important role of routine hospital-level data in the assessment of the opioid burden, nationally. Policymakers should link data sources, such as death registries, hospital data and prescription data to understand causes and consequences of opioid use disorders along the patient pathway.

### Policy implications and conclusion

Despite progress in addressing opioid-related mortality in England, the detrimental effects of harmful opioid use on population health, the National Health Service and public finances remain substantial. Opioid-related hospitalisations increased by 48.9% across the 10-year study period, and we observed marked rises in hospitalisations between 2010 and 2013, mostly driven by opioid poisoning. This could be an effect caused by government prescribed fiscal consolidation that resulted in cuts to welfare support, causing rising levels of unemployment,^28^ poverty and its correlates such as homelessness.^29^ Death of despair may be one reason for rises in opioid mortality and admissions; however, the increase in opioid-related hospitalisations was about 40% higher than those due to alcohol or other illicit drugs. It is also possible that some of the observed increases in opioid misuse and poisoning may reflect changes in opioid prescriptions in England. Between 1998 and 2016, opioid prescriptions increased by 127%, from 190,000 mg to 431,000 mg per 1000 population.^30^ Even though prescribing trends have flattened in the past five years, the strength of prescribed opiates drugs had increased successively.

Based on our findings, adverse effects of opioids appear to play an increasingly important role within older, less deprived and sicker population groups. This study highlights the need for a more systematic approach to target people at risk from harmful use of opioids. Administrative hospital data may be a useful tool to identify individuals struggling with opioids, help target prevention programmes and measure their impact beyond official mortality statistics.

## Supplemental Material

sj-pdf-1-jrs-10.1177_01410768221077360 - Supplemental material for Trends and characteristics of hospitalisations from the harmful use of opioids in England between 2008 and 2018: Population-based retrospective cohort study Click here for additional data file. Supplemental material, sj-pdf-1-jrs-10.1177_01410768221077360 for Trends and characteristics of hospitalisations from the harmful use of opioids in England between 2008 and 2018: Population-based retrospective cohort study by Rocco Friebel and Laia Maynou in Journal of the Royal Society of Medicine

sj-pdf-2-jrs-10.1177_01410768221077360 - Supplemental material for Trends and characteristics of hospitalisations from the harmful use of opioids in England between 2008 and 2018: Population-based retrospective cohort study Click here for additional data file. Supplemental material, sj-pdf-2-jrs-10.1177_01410768221077360 for Trends and characteristics of hospitalisations from the harmful use of opioids in England between 2008 and 2018: Population-based retrospective cohort study by Rocco Friebel and Laia Maynou in Journal of the Royal Society of Medicine
