# Supplementary material for: Trends and characteristics of hospitalisations from the harmful use of opioids in England between 2008 and 2018: Population-based retrospective cohort study
Source: J R Soc Med. 2022 Feb 3;115(5):173–85. doi: 10.1177/01410768221077360 (PMC9066666; doi:10.1177/01410768221077360)
Supplement: sj-pdf-1-jrs-10.1177_01410768221077360 - Supplemental material for Trends and characteristics of hospitalisations from the harmful use of opioids in England between 2008 and 2018: Population-based retrospective cohort study [file sj-pdf-1-jrs-10.1177_01410768221077360.pdf]

Appendix A. ICD-10 codes for opioid-related hospitalisations

Poisoning heroin:
T401X1A
T401X2A
T401X3A
T401X4A

Poisoning:
F1110
T402X1A
T402X2A
T402X3A
T402X4A
T404X1A
T404X2A
T404X3A
T404X4A
T40601A
T40602A
T40603A
T40604A
T40691A
T40692A
T40693A
T40694A
T400X1A
T400X2A
T400X3A
T400X4A
T403X1A
T403X2A
T403X3A
T403X4A

Dependence/abuse:
F1120
F1121
F1920
