# Supplementary material for: Trends and characteristics of hospitalisations from the harmful use of opioids in England between 2008 and 2018: Population-based retrospective cohort study
Source: J R Soc Med. 2022 Feb 3;115(5):173–85. doi: 10.1177/01410768221077360 (PMC9066666; doi:10.1177/01410768221077360)
Supplement: sj-pdf-2-jrs-10.1177_01410768221077360 - Supplemental material for Trends and characteristics of hospitalisations from the harmful use of opioids in England between 2008 and 2018: Population-based retrospective cohort study [file sj-pdf-2-jrs-10.1177_01410768221077360.pdf]

Appendix B

Table A1. Regression output of the association between patient characteristics and In-hospital mortality per year

**Dep. var. In-hospital mortality (logistic regression)**	**(1)**	**(2)**	**(3)**	**(4)**	**(5)**	**(6)**	**(7)**	**(8)**	**(9)**	**(10)**	**(11)**
	**2008**	**2009**	**2010**	**2011**	**2012**	**2013**	**2014**	**2015**	**2016**	**2017**	**2018**
**Female**	Reference	Reference	Reference	Reference	Reference	Reference	Reference	Reference	Reference	Reference	Reference
**Male**	1.503	1.047	1.153	1.509	2.816**	3.057***	2.054**	2.284***	1.667*	1.804**	1.451
	(0.480)	(0.309)	(0.427)	(0.484)	(1.147)	(0.964)	(0.611)	(0.555)	(0.449)	(0.500)	(0.376)
**Age 0-14**	Reference	Reference	Reference	Reference	Reference	Reference	Reference	Reference	Reference	Reference	Reference
**Age 15-24**	0.062***		0.136**	1.371			0.078**	0.034***	0.052**	0.222*	0.161
	(0.052)		(0.121)	(1.717)			(0.088)	(0.038)	(0.060)	(0.203)	(0.228)
**Age 25-34**	0.078***	0.092***	0.107**	1.433	0.598	0.151***	0.460	0.138***	0.278*	0.886	0.514
	(0.061)	(0.069)	(0.096)	(1.719)	(0.575)	(0.107)	(0.295)	(0.088)	(0.184)	(0.560)	(0.576)
**Age 35-44**	0.064***	0.086***	0.165**	1.875	0.324	0.083***	0.413	0.447*	0.468	0.539	1.297
	(0.050)	(0.062)	(0.133)	(2.120)	(0.312)	(0.062)	(0.268)	(0.219)	(0.270)	(0.348)	(1.368)
**Age 45-54**	0.096***	0.182**	0.107**	1.331	0.299	0.166***	0.507	0.183***	0.245**	0.527	1.433
	(0.075)	(0.124)	(0.099)	(1.532)	(0.291)	(0.103)	(0.311)	(0.098)	(0.143)	(0.319)	(1.544)
**Age 55-64**	0.302	0.377	0.480	4.070	0.568	0.224**	0.474	0.291**	0.277**	0.630	1.306
	(0.223)	(0.240)	(0.406)	(4.523)	(0.519)	(0.151)	(0.311)	(0.155)	(0.158)	(0.359)	(1.469)
**Age 65-74**	0.504	0.713	1.156	3.113	0.499	0.266**	0.520	0.746	0.180**	0.153**	1.592
	(0.346)	(0.432)	(0.944)	(3.528)	(0.476)	(0.169)	(0.347)	(0.337)	(0.121)	(0.127)	(1.809)
**Age 75-84**	0.692	1.663	2.179	1.633	1.829	0.800	1.203	0.506	0.322*	1.213	3.656
	(0.465)	(0.888)	(1.708)	(1.920)	(1.500)	(0.418)	(0.733)	(0.274)	(0.189)	(0.633)	(4.065)

**Age 85+**											3.288
											(3.737)
**Charlson Comorbidity Index =0**	Reference	Reference	Reference	Reference	Reference	Reference	Reference	Reference	Reference	Reference	Reference
**Charlson Comorbidity Index =1**	2.036	1.497	1.422	1.753	2.607**	1.151	2.716***	2.498***	3.276***	3.040***	2.531***
	(0.968)	(0.712)	(0.709)	(0.891)	(1.233)	(0.565)	(0.974)	(0.746)	(1.143)	(1.143)	(0.906)
**Charlson Comorbidity Index =2**	2.534	2.591*	1.664	10.51***	1.608	2.950**	3.241**	2.400*	5.032***	6.524***	2.597**
	(1.587)	(1.376)	(1.105)	(5.081)	(1.389)	(1.437)	(1.679)	(1.078)	(2.278)	(3.073)	(1.242)
**Charlson Comorbidity Index =3**	3.538	6.734***	2.126	20.56***		4.261**	7.221***	2.920*	5.085**	13.64***	5.439***
	(2.750)	(3.864)	(1.691)	(13.29)		(2.710)	(3.980)	(1.696)	(3.271)	(6.937)	(2.872)
**Charlson Comorbidity Index =4**		4.152*		6.496*	12.43***	3.495	7.532***	7.298***	7.825***	8.255***	4.142**
		(3.245)		(6.826)	(10.54)	(3.104)	(5.352)	(4.017)	(5.887)	(5.844)	(2.696)
**Charlson Comorbidity Index =5**	35.79***	5.500	9.950***	25.25***	12.83**	4.174		9.463***			4.656*
	(29.72)	(6.431)	(8.158)	(28.63)	(16.25)	(5.004)		(7.186)			(3.731)
**Charlson Comorbidity Index =6+**	1.571	7.375***	2.061	24.68***	7.583**	4.760**	9.093***	9.644***	28.58***	17.15***	5.349***
	(1.683)	(3.526)	(1.762)	(15.80)	(6.431)	(3.112)	(5.611)	(4.643)	(14.73)	(10.18)	(3.180)
**IMD quintile 5 - Most deprived**	1.261	0.787	0.642	0.386**	1.091	0.609	0.935	0.782	1.516	0.958	1.203
	(0.858)	(0.604)	(0.330)	(0.175)	(0.801)	(0.286)	(0.458)	(0.343)	(0.870)	(0.509)	(0.577)
**IMD quintile 4**	1.454	2.882	0.447	0.319**	1.035	0.410*	0.648	0.982	2.345	0.970	1.310
	(1.018)	(1.871)	(0.263)	(0.161)	(0.781)	(0.213)	(0.357)	(0.417)	(1.334)	(0.550)	(0.630)
**IMD quintile 3**	0.689	2.208	0.669	0.414*	1.070	0.727	0.777	0.744	2.351	1.428	0.993
	(0.512)	(1.484)	(0.362)	(0.199)	(0.812)	(0.344)	(0.418)	(0.338)	(1.336)	(0.766)	(0.501)
**IMD quintile 2**	1.853	1.978	0.389	0.582	0.982	0.522	1.816	1.290	1.102	2.213	1.319
	(1.293)	(1.371)	(0.281)	(0.299)	(0.780)	(0.304)	(0.926)	(0.587)	(0.730)	(1.170)	(0.674)
**IMD quintile 1 - Less deprived**	Reference	Reference	Reference	Reference	Reference	Reference	Reference	Reference	Reference	Reference	Reference
**Number previous admissions**	0.398	0.530	0.524	1.032	0.694	0.775	0.817	0.770*	0.558**	0.720*	0.606**
	(0.348)	(0.281)	(0.289)	(0.0541)	(0.167)	(0.137)	(0.130)	(0.113)	(0.135)	(0.128)	(0.119)

**N**	9,888	8,623	11,140	10,879	10,955	12,646	14,961	14,921	13,750	13,809	14,966
**Pseudo R-squared**	0.162	0.230	0.141	0.171	0.098	0.133	0.108	0.130	0.132	0.130	0.107
**Hospital FE**	No	No	No	No	No	No	No	No	No	No	No

Note: Significance levels: *** p<0.01, ** p<0.05, * p<0.1; Odds ratios reported and standard errors reported in parenthesis.

Table A2. Regression output of the association between patient characteristics and LoS per year

**Dep. var.: LoS (OLS)**	**(1)**	**(2)**	**(3)**	**(4)**	**(5)**	**(6)**	**(7)**	**(8)**	**(9)**	**(10)**	**(11)**
	**2008**	**2009**	**2010**	**2011**	**2012**	**2013**	**2014**	**2015**	**2016**	**2017**	**2018**
**Female**	Reference	Reference	Reference	Reference	Reference	Reference	Reference	Reference	Reference	Reference	Reference
**Male**	0.020	-0.031	-0.003	-0.065	-0.072	-0.133***	-0.067	-0.012	-0.033	0.004	-0.067**
	(0.071)	(0.067)	(0.0663)	(0.063)	(0.050)	(0.045)	(0.043)	(0.042)	(0.041)	(0.039)	(0.034)
**Age 0-14**	Reference	Reference	Reference	Reference	Reference	Reference	Reference	Reference	Reference	Reference	Reference
**Age 15-24**	-0.396***	-0.554***	-0.331***	-0.372***	-0.173*	-0.113	-0.106*	-0.114	-0.161*	-0.0854	-0.211**
	(0.092)	(0.107)	(0.103)	(0.114)	(0.103)	(0.072)	(0.060)	(0.081)	(0.084)	(0.073)	(0.102)
**Age 25-34**	-0.372***	-0.327***	-0.199*	-0.318**	-0.178	-0.0641	-0.0969	-0.222***	-0.143	-0.0673	-0.174*
	(0.101)	(0.115)	(0.119)	(0.124)	(0.109)	(0.077)	(0.066)	(0.084)	(0.088)	(0.074)	(0.105)
**Age 35-44**	-0.183*	-0.212*	-0.0867	-0.219*	-0.00694	0.00647	-0.00671	-0.0232	-0.096	-0.028	-0.039
	(0.102)	(0.116)	(0.116)	(0.126)	(0.111)	(0.080)	(0.070)	(0.088)	(0.088)	(0.077)	(0.107)
**Age 45-54**	0.0108	-0.116	-0.097	-0.076	0.0533	0.139*	0.0774	0.0263	-0.0131	0.115	-0.0967
	(0.127)	(0.126)	(0.121)	(0.135)	(0.114)	(0.082)	(0.073)	(0.091)	(0.090)	(0.081)	(0.107)
**Age 55-64**	0.221	0.331*	0.419***	0.158	0.376***	0.281***	0.208**	0.393***	0.234**	0.172*	0.0529
	(0.173)	(0.170)	(0.157)	(0.159)	(0.136)	(0.108)	(0.094)	(0.117)	(0.109)	(0.093)	(0.116)

**Age 65-74**	1.618***	0.861***	0.389**	0.645***	0.788***	0.667***	0.602***	0.455***	0.349**	0.518***	0.317**
	(0.333)	(0.280)	(0.189)	(0.239)	(0.208)	(0.184)	(0.169)	(0.158)	(0.146)	(0.138)	(0.149)
**Age 75-84**	1.754***	1.897***	2.104***	0.937***	1.370***	1.242***	0.770***	1.108***	1.059***	0.769***	0.466**
	(0.416)	(0.423)	(0.407)	(0.308)	(0.298)	(0.270)	(0.218)	(0.255)	(0.243)	(0.196)	(0.187)
**Age 85+**	3.439***	1.643***	1.239**	1.943***	1.148***	1.889***	1.100***	1.421***	1.264***	1.000***	0.372
	(0.779)	(0.540)	(0.497)	(0.523)	(0.326)	(0.434)	(0.312)	(0.367)	(0.338)	(0.313)	(0.236)
**Charlson Comorbidity Index =0**	Reference	Reference	Reference	Reference	Reference	Reference	Reference	Reference	Reference	Reference	Reference
**Charlson Comorbidity Index =1**	0.422***	0.233***	0.253***	0.271***	0.157**	0.255***	0.133**	0.178***	0.125**	0.182***	0.148***
	(0.118)	(0.089)	(0.091)	(0.077)	(0.065)	(0.064)	(0.055)	(0.058)	(0.056)	(0.051)	(0.045)
**Charlson Comorbidity Index =2**	0.584**	0.543*	0.243	0.975***	0.577***	0.320**	0.658***	0.373***	0.136	0.282**	0.258***
	(0.285)	(0.301)	(0.216)	(0.238)	(0.206)	(0.139)	(0.153)	(0.137)	(0.116)	(0.117)	(0.098)
**Charlson Comorbidity Index =3**	2.157***	1.086**	0.542	1.106**	1.234***	0.926***	0.532**	0.321	0.751***	0.549***	0.348*
	(0.727)	(0.531)	(0.424)	(0.487)	(0.413)	(0.290)	(0.231)	(0.227)	(0.248)	(0.209)	(0.191)
**Charlson Comorbidity Index =4**	2.901**	1.047	2.009**	2.363***	0.899	2.069***	1.262***	1.013**	0.599	0.802**	0.427*
	(1.287)	(1.061)	(0.878)	(0.766)	(0.570)	(0.654)	(0.468)	(0.466)	(0.379)	(0.354)	(0.233)
**Charlson Comorbidity Index =5**	7.592***	-0.0809	3.950**	2.812	1.167	2.031*	2.300*	1.595**	0.311	0.368	0.667
	(2.626)	(1.407)	(1.649)	(1.836)	(0.992)	(1.204)	(1.176)	(0.626)	(0.501)	(0.355)	(0.542)
**Charlson Comorbidity Index =6+**	0.777	1.170**	1.250**	0.802	0.149	0.653*	1.278***	0.579	0.403	0.652*	0.241
	(0.537)	(0.574)	(0.502)	(0.551)	(0.315)	(0.341)	(0.399)	(0.371)	(0.266)	(0.373)	(0.206)
**IMD quintile 5 - Most deprived**	-0.302*	-0.090	0.027	-0.162	0.0217	0.012	-0.113	-0.021	-0.112	-0.052	0.003
	(0.155)	(0.140)	(0.132)	(0.130)	(0.096)	(0.090)	(0.088)	(0.088)	(0.083)	(0.078)	(0.060)
**IMD quintile 4**	-0.113	-0.165	0.039	-0.232*	-0.100	0.0231	-0.107	0.0198	-0.067	-0.073	0.034
	(0.157)	(0.142)	(0.134)	(0.132)	(0.097)	(0.093)	(0.086)	(0.090)	(0.086)	(0.078)	(0.058)
**IMD quintile 3**	-0.272*	-0.019	-0.181	-0.170	-0.035	0.037	-0.103	0.019	-0.087	-0.025	0.032
	(0.158)	(0.144)	(0.134)	(0.136)	(0.101)	(0.092)	(0.087)	(0.086)	(0.084)	(0.079)	(0.063)
**IMD quintile 2**	-0.120	-0.243	0.047	-0.127	0.119	0.120	-0.077	-0.011	0.014	0.006	0.029
	(0.173)	(0.151)	(0.148)	(0.153)	(0.115)	(0.105)	(0.099)	(0.095)	(0.093)	(0.086)	(0.067)

**IMD quintile 1 - Less deprived**	Reference	Reference	Reference	Reference	Reference	Reference	Reference	Reference	Reference	Reference	Reference
**Number previous admissions**	-0.075	-0.039	-0.009	-0.028*	-0.021	-0.014	-0.016	-0.022**	0.011	0.005	-0.019***
	(0.048)	(0.030)	(0.027)	(0.014)	(0.016)	(0.015)	(0.013)	(0.011)	(0.009)	(0.010)	(0.004)
**N**	10,077	10,873	11,384	11,040	13,956	15,896	15,292	15,143	14,010	14,100	14,810
**R-squared**	0.565	0.610	0.608	0.611	0.538	0.550	0.570	0.439	0.419	0.379	0.378
**Hospital FE**	Yes	Yes	Yes	Yes	Yes	Yes	Yes	Yes	Yes	Yes	Yes

Note: Significance levels: *** p<0.01, ** p<0.05, * p<0.1; Marginal effects reported and standard errors reported in parenthesis; OLS – ordinary least squares. Patients who died are not included.

Table A3. Regression output of the association between patient characteristics and 30-day readmission per year

**Dep. var.: 30-day readmission (logistic regression)**	**(1)**	**(2)**	**(3)**	**(4)**	**(5)**	**(6)**	**(7)**	**(8)**	**(9)**	**(10)**	**(11)**
	**2008**	**2009**	**2010**	**2011**	**2012**	**2013**	**2014**	**2015**	**2016**	**2017**	**2018**
**Female**	Reference	Reference	Reference	Reference	Reference	Reference	Reference	Reference	Reference	Reference	Reference
**Male**	0.984	0.987	1.075	1.153***	1.074	1.089*	1.061	0.978	1.036	1.158***	1.030
	(0.060)	(0.057)	(0.061)	(0.063)	(0.051)	(0.049)	(0.048)	(0.045)	(0.048)	(0.055)	(0.047)
**Age 0-14**	Reference	Reference	Reference	Reference	Reference	Reference	Reference	Reference	Reference	Reference	Reference
**Age 15-24**	1.760**	1.829**	1.833**	2.364***	2.291***	2.068***	1.477**	2.885***	1.505**	1.955***	1.395*
	(0.473)	(0.508)	(0.448)	(0.635)	(0.500)	(0.412)	(0.274)	(0.692)	(0.275)	(0.409)	(0.253)
**Age 25-34**	2.287***	2.465***	1.972***	2.651***	2.092***	2.278***	1.810***	2.834***	1.584**	2.214***	1.585**
	(0.609)	(0.681)	(0.481)	(0.708)	(0.457)	(0.453)	(0.331)	(0.680)	(0.287)	(0.461)	(0.286)
**Age 35-44**	2.782***	3.110***	2.260***	2.724***	2.463***	2.185***	1.873***	3.340***	1.440**	2.196***	1.511**
	(0.738)	(0.855)	(0.549)	(0.727)	(0.536)	(0.435)	(0.341)	(0.800)	(0.262)	(0.457)	(0.273)

**Age 45-54**	2.575***	3.271***	2.370***	2.674***	2.350***	2.463***	1.908***	3.095***	1.517**	1.866***	1.484**
	(0.701)	(0.912)	(0.582)	(0.723)	(0.516)	(0.492)	(0.350)	(0.744)	(0.277)	(0.392)	(0.270)
**Age 55-64**	2.844***	3.272***	2.751***	2.803***	2.811***	2.355***	1.978***	3.210***	1.436*	1.975***	1.097
	(0.819)	(0.947)	(0.712)	(0.791)	(0.639)	(0.497)	(0.384)	(0.796)	(0.275)	(0.428)	(0.210)
**Age 65-74**	3.061***	2.587***	2.505***	1.814*	2.452***	2.269***	2.060***	3.506***	1.309	2.458***	1.478*
	(0.950)	(0.833)	(0.700)	(0.560)	(0.602)	(0.523)	(0.428)	(0.904)	(0.274)	(0.559)	(0.297)
**Age 75-84**	3.499***	2.633***	2.663***	3.312***	2.559***	2.117***	1.961***	2.919***	2.056***	2.507***	1.369
	(1.123)	(0.850)	(0.758)	(1.012)	(0.657)	(0.516)	(0.435)	(0.794)	(0.441)	(0.602)	(0.295)
**Age 85+**	3.001***	2.460**	3.118***	3.220***	3.308***	2.422***	1.827**	3.295***	2.077***	2.657***	1.246
	(1.116)	(0.909)	(0.999)	(1.084)	(0.895)	(0.630)	(0.453)	(0.930)	(0.479)	(0.677)	(0.299)
**Charlson Comorbidity Index =0**	Reference	Reference	Reference	Reference	Reference	Reference	Reference	Reference	Reference	Reference	Reference
**Charlson Comorbidity Index =1**	1.206**	1.238***	1.395***	1.492***	1.352***	1.447***	1.368***	1.288***	1.311***	1.284***	1.581***
	(0.110)	(0.099)	(0.105)	(0.105)	(0.084)	(0.082)	(0.078)	(0.074)	(0.077)	(0.076)	(0.088)
**Charlson Comorbidity Index =2**	1.557**	1.906***	1.574***	2.142***	2.046***	1.870***	1.424***	1.799***	1.861***	1.693***	2.247***
	(0.274)	(0.282)	(0.223)	(0.283)	(0.225)	(0.193)	(0.144)	(0.173)	(0.179)	(0.168)	(0.205)
**Charlson Comorbidity Index =3**	2.361***	1.968***	2.387***	1.553*	2.075***	2.341***	1.832***	2.061***	2.266***	1.981***	2.392***
	(0.613)	(0.470)	(0.512)	(0.357)	(0.355)	(0.377)	(0.280)	(0.286)	(0.301)	(0.283)	(0.306)
**Charlson Comorbidity Index =4**	1.392	2.199*	3.485***	1.759*	1.725**	2.293***	1.398	3.064***	2.938***	2.502***	2.181***
	(0.634)	(0.887)	(1.144)	(0.557)	(0.474)	(0.534)	(0.333)	(0.626)	(0.545)	(0.456)	(0.401)
**Charlson Comorbidity Index =5**	1.490	13.64***	1.291	0.861	0.863	2.039*	3.737***	2.340***	3.074***	2.220***	2.940***
	(0.967)	(7.582)	(0.749)	(0.697)	(0.559)	(0.843)	(1.374)	(0.667)	(0.906)	(0.640)	(0.734)
**Charlson Comorbidity Index =6+**	1.671*	2.107***	2.604***	2.084***	2.241***	1.450*	1.739**	2.236***	1.392	1.885***	2.459***
	(0.519)	(0.524)	(0.630)	(0.501)	(0.517)	(0.320)	(0.379)	(0.446)	(0.295)	(0.357)	(0.453)
**IMD quintile 5 - Most deprived**	1.135	1.087	0.994	1.149	1.193*	1.130	1.149	1.131	1.373***	1.107	1.212**
	(0.154)	(0.132)	(0.117)	(0.140)	(0.120)	(0.103)	(0.111)	(0.109)	(0.130)	(0.104)	(0.110)
**IMD quintile 4**	1.173	1.091	1.014	1.103	1.253**	0.993	1.227**	1.146	1.183*	1.134	1.051
	(0.161)	(0.133)	(0.120)	(0.136)	(0.128)	(0.092)	(0.118)	(0.110)	(0.116)	(0.107)	(0.096)

**IMD quintile 3**	1.060	1.011	0.878	1.166	1.221*	1.003	1.263**	1.207*	1.200*	1.188*	1.076
	(0.146)	(0.126)	(0.106)	(0.145)	(0.125)	(0.093)	(0.122)	(0.116)	(0.117)	(0.112)	(0.099)
**IMD quintile 2**	1.061	1.094	0.907	1.023	0.983	0.844	1.129	1.056	1.100	0.988	1.003
	(0.160)	(0.146)	(0.121)	(0.139)	(0.113)	(0.089)	(0.121)	(0.111)	(0.117)	(0.103)	(0.100)
**IMD quintile 1 - Less deprived**	Reference	Reference	Reference	Reference	Reference	Reference	Reference	Reference	Reference	Reference	Reference
**Number previous admissions**	2.405***	1.639***	1.694***	1.521***	1.467***	1.455***	1.355***	1.413***	1.283***	1.281***	1.256***
	(0.197)	(0.086)	(0.077)	(0.076)	(0.052)	(0.035)	(0.028)	(0.035)	(0.026)	(0.027)	(0.035)
**N**	9,909	10,784	11,340	11,033	14,013	15,976	15,388	15,207	14,111	14,218	14,913
**R-squared**	0.091	0.091	0.083	0.075	0.067	0.073	0.066	0.076	0.065	0.064	0.074
**Hospital FE**	Yes	Yes	Yes	Yes	Yes	Yes	Yes	Yes	Yes	Yes	Yes

Note: Significance levels: *** p<0.01, ** p<0.05, * p<0.1; Odds ratios reported and standard errors reported in parenthesis.
